# Preoperative prediction of serosal invasion in gastric cancer using multiphase contrast-enhanced CT radiomics

**DOI:** 10.3389/fonc.2026.1879678

**Published:** 2026-09-15

**Authors:** Xiaojiao Li, Chencui Huang, Teng Ma, Liguang Wang

**Affiliations:** 1 Department of Radiology, Shandong Provincial Hospital Affiliated to Shandong First Medical University, Jinan, Shandong, China; 2 Department of Research Collaboration, Research and Development (R&D) Center, Hangzhou Deepwise & League of Precision Health Diagnostics (PHD) Technology Co., Ltd, Hangzhou, Zhejiang, China; 3 Department of Oncology, Shandong Provincial Hospital Affiliated to Shandong First Medical University, Jinan, Shandong, China

**Keywords:** Ca724, contrast-enhanced CT, gastric cancer, nomogram, preoperative prediction, radiomics, serosal invasion

## Abstract

**Objective:**

This study aimed to develop and validate a radiomics-based model using multiphase contrast-enhanced CT for the preoperative prediction of serosal invasion in gastric cancer, and to evaluate whether the integration of clinical and CT morphological features could further improve predictive performance.

**Methods:**

A retrospective analysis was conducted on 183 patients with surgically confirmed gastric cancer. All patients underwent multiphase CT (arterial, venous, and delayed phases). Radiomic features were extracted from volumetric regions of interest (VOIs). After feature selection and radiomics model construction, a combined nomogram was developed incorporating independent clinical and imaging predictors. Models were evaluated in training and validation cohorts using AUC, accuracy, sensitivity, and specificity.

**Results:**

The arterial plus delayed (AP+DP) phase radiomics model demonstrated the best performance, with AUCs of 0.8256 and 0.8179 in the training and validation sets, respectively. Multivariate analysis identified serum CA724 levels and serosal stranding as independent predictors. The combined nomogram incorporating these factors and the radiomics signature achieved AUCs of 0.8626 and 0.8641 in the training and validation sets, respectively. A clinical model was also established, with AUCs of 0.7738 and 0.7654 for the training and validation sets.

**Conclusion:**

A radiomics model based on AP+DP CT phases shows strong potential for predicting serosal invasion in GC.The integration of CA72-4 and serosal stranding further increased the AUC of the combined model. The combined model achieved a statistically significant improvement in AUC over the clinical model in the training set, providing a non-invasive tool to support preoperative decision making.

## 1 Introduction

Gastric cancer (GC) is the fifth most common cancer globally and a leading cause of cancer-related death, particularly in East Asia (1). The majority of cases are diagnosed at an advanced stage. Accurate TNM staging is essential in current clinical practice to guide treatment planning and inform decisions regarding surgery or other medical interventions (2). The outermost layer of the gastric wall, the serosa, serves as a defensive barrier against tumor dissemination. Invasion of the serosa increases the risk of peritoneal metastasis (PM) in GC patients (3, 4). Those with peritoneal metastases have a poor prognosis, with a median survival of ≤1 year and low five-year survival rates (5). The National Comprehensive Cancer Network (NCCN) 2016 clinical guidelines recommend neoadjuvant chemotherapy (NAC) for patients with T4 advanced GC to reduce tumor stage, improve the R0 resection rate, and enhance overall survival (6).

Computed tomography (CT) and endoscopic ultrasound (EUS) are the primary imaging techniques used for GC staging. However, EUS is an operator-dependent invasive procedure associated with sedation-related risks and limited accessibility to certain gastric regions-such as the lesser curvature, subcardiac area, and gastroesophageal junction-as well as difficulties in evaluating extensive ulcerative or large lesions (7). Advancements in CT technology have strengthened its role in both T and N staging. The accuracy of T staging can be influenced by tumor location, the extent and width of the bare area of the stomach, and conditions within the perigastric fat space. Reported accuracy rates of CT in diagnosing serosal invasion range from 55.9% to 90.8% (8–11), while post-NAC T restaging accuracy drops to around 57% (12). Therefore, improving diagnostic accuracy for serosal invasion is crucial for preoperative and prognostic evaluation.

Radiomics aims to extract quantitative and reproducible information from medical images—including patterns imperceptible to the human eye—to characterize tissue and lesion properties (e.g., shape and heterogeneity) and monitor changes over time or in response to treatment (13). Studies have shown that radiomic features strongly correlate with cellular-level heterogeneity indices (14, 15) and tumor aggressiveness (16). In recent years, radiomic studies have increasingly focused on predicting histological subtypes, lymph node metastasis, lymphatic invasion, peritoneal metastasis, and response to NAC in GC (17–21). Several studies have explored CT-based radiomics for predicting serosal invasion in GC, often using single-phase whole-tumor volume of interest (VOI) or multi-phase single-slice region of interest (ROI) models. These include both pure radiomic models and combined models integrating clinical and radiomic features (22–25). For instance, Wang et al. (26) used portal venous phase and iodine map images to predict serosal invasion after NAC. However, important questions remain: whether multi-phase CT offers advantages over single-phase imaging in predicting serosal invasion, and whether three-dimensional VOI-based segmentation improves prediction accuracy.

This study aims to develop a predictive model integrating multi-phase contrast-enhanced CT radiomics with CT morphological features and clinical data to evaluate the extent of serosal invasion in GC, thereby improving preoperative T staging and prognostic assessment.

## 2 Methods

### 2.1 Patients

A retrospective analysis was conducted on patients pathologically diagnosed with GC via surgical resection at our institution between April 2018 and June 2019. All patients underwent contrast-enhanced CT scans within two weeks before surgery. This study was approved by the Institutional Ethics Committee (Approval No.: SWYX: NO.2023-440).

Inclusion criteria were as follows: (1) Surgical confirmation of GC with clear pathological T-stage; (2) Completion of abdominal contrast-enhanced CT including arterial (AP), venous (VP), and delayed phases (DP) within two weeks before surgery, with images suitable for evaluation; (3) Availability of complete clinical and laboratory data.Based on postoperative pathological T-stage, patients were categorized into serosal invasion-positive (T4) and serosal invasion-negative (T1–3) groups. They were randomly divided into training (n=128) and validation (n=55) sets at a 7:3 ratio. CT characteristics and clinical data were recorded, including sex, age, tumor location [cardia (C), body (B), antrum (A)], presence of ulceration, continuity of the mucosal line, serosal surface irregularity with strand-like changes (>1/3 of lesion volume), invasive growth pattern, lesion thickness, presence of enlarged lymph nodes (short-axis ≥1 cm or extra-gastric node ≥8 mm), and levels of CA724 and CA199.

### 2.2 CT scanning protocol

All patients fasted for >8 hours and consumed 600–1000 ml of water before the scan. Contrast-enhanced abdominal CT was performed using scanners (Aquilion ONE, Toshiba; Ingenuity CT, Philips; Somatom Definition Flash/Force, Siemens) covering the entire gastric region. Scanning parameters: tube voltage 120 kV, tube current 250–400 mA, pitch 1.388 or 0.984, matrix 512×512, detector collimation 80×0.5 mm or 60×0.625 mm, rotation time 0.5 s, slice thickness 5 mm. Nonionic iodinated contrast (Omnipaque 350, GE Healthcare) was injected via the antecubital vein at 2.5–3.0 ml/s using a high-pressure injector (Ulrich CT Plus 150), with a total iodine dose of 400–450 mg/kg. AP, VP, and DP scans were acquired at 22–30 s, 60–70 s, and 120–130 s, respectively.

### 2.3 CT feature analysis

Two radiologists (with 10 and 15 years of abdominal imaging experience) independently and blindly evaluated AP, VP, and DP images without knowledge of pathological or laboratory results. Assessed features included: Tumor location [non-cardia (B/A) = 0, cardia (C) = 1]; Ulceration (present = 1, absent = 0); Mucosal line continuity (interrupted = 1, continuous/thickened = 0); Serosal Stranding (>1/3 lesion volume = 1, ≤1/3 = 0); Invasive growth (ill-defined border = 1, well-defined = 0); Lesion thickness (mean of two measurements at largest cross-section); Enlarged lymph nodes (present = 1, absent = 0). CT feature assessment is shown in **Figures 1**, **2**.

**Figure 1:**
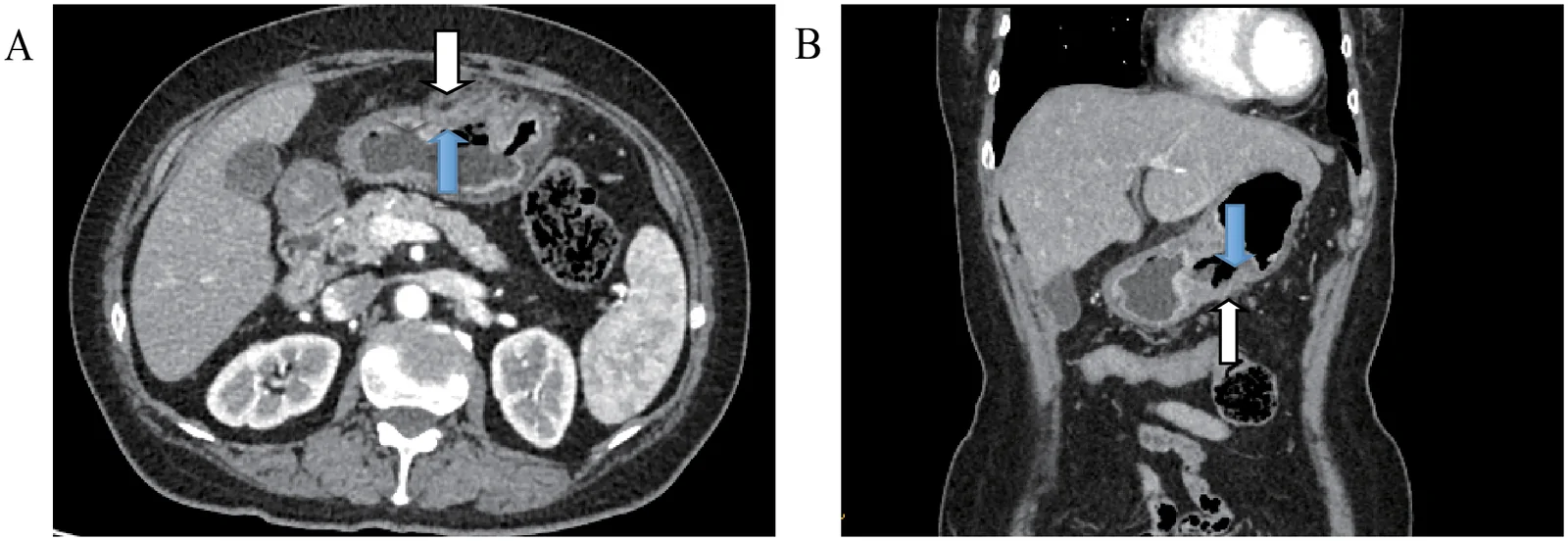
**(A, B)** show axial and coronal images from the arterial phase, respectively. White arrows indicate serosal surface irregularity with strand-like changes and haziness of the surrounding fat space. Blue arrows demonstrate local ulceration with interrupted mucosal line discontinuity. The tumor exhibits infiltrative growth with poorly defined margins.

**Figure 2:**
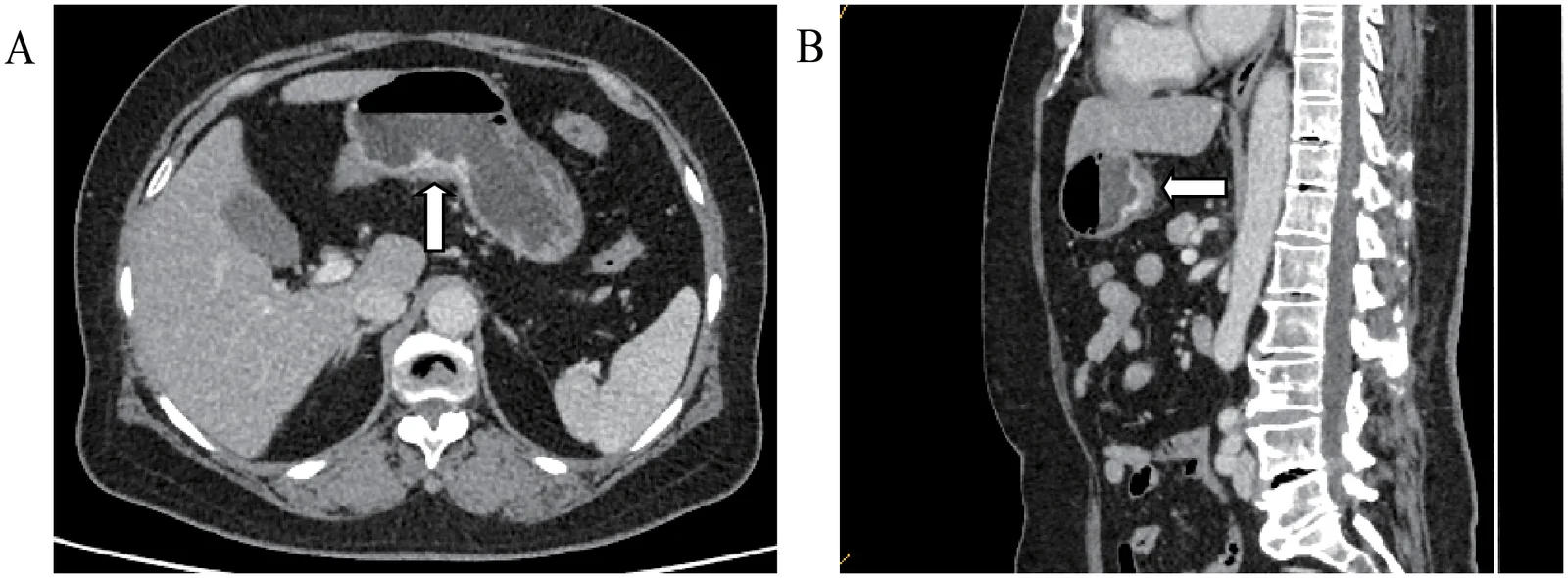
**(A, B)** show axial and sagittal images from the venous phase, respectively. White arrows indicate a few short strand-like changes on the serosal surface. The gastric wall shows localized thickening with ulceration on the superficial aspect, while the mucosa remains continuous and demonstrates marked enhancement.

CA199 and CA724 were dichotomized (CA199: >39 U/ml = 1, ≤39 U/ml = 0; CA724: >6.9 U/ml = 1, ≤6.9 U/ml = 0). Continuous variables were averaged between readers; discrepancies in categorical variables were resolved through consensus.

### 2.4 Tumor segmentation and radiomics feature extraction/selection

DICOM images (AP/VP/DP) were uploaded to the DeepWise Multimodal Research Platform V2.3.1 (Beijing Deepwise & League of PhD Technology Co., Ltd.). Reader A semi-automatically segmented each slice along tumor margins to generate a 3D VOI, avoiding peritumoral gas/vessels/fat. VOIs were labeled based on pathological T-stage (T1–3: serosal-negative, T4: serosal-positive).

Images were resampled to an isotropic resolution of [1, 1, 1] via B-spline interpolation. Feature normalization was performed using z-scoring based on parameters (mean ± SD) derived from the training set. Features with inter- or intra-observer ICC < 0.9 were excluded. Correlation filtering with a threshold of 0.8 was subsequently applied to eliminate redundant features.

### 2.5 Radiomics model construction

The dataset was split 7:3 into training and validation sets. Models were built using various feature selection methods and modeling algorithms. Rad-scores were calculated for each patient. Models were developed for single-phase (AP, VP, DP) and multi-phase combinations (AP+VP, AP+DP, VP+DP, AP+VP+DP). The best-performing model was integrated with CT/clinical features into a nomogram using logistic regression (LR).

### 2.6 Model validation and evaluation

ROC analysis assessed diagnostic performance (AUC, accuracy, sensitivity, specificity). Delong’s test compared AUCs among themodels,.

### 2.7 Statistical analysis

Statistical analyses were performed in SPSS 23. Continuous normally distributed variables (confirmed by KS/SW test, p > 0.05) were reported as mean ± SD and compared via t-test; non-normal variables via Mann-Whitney test. Categorical variables were presented as frequencies/percentages and compared using chi-square or Fisher’s exact test. Univariate and multivariate logistic regression identified independent predictors. DeLong’s test was used to evaluate differences in model performance, and *p*-values were adjusted via the Holm-Bonferroni method for multiple comparisons. Model calibration was assessed by Brier scores and the Hosmer-Lemeshow goodness-of-fit test. A lower Brier score indicates better calibration. Non-significant *p*-value of the Hosmer-Lemeshow test suggests acceptable model fit. Calibration curves were plotted for visual evaluation. Decision-curve analysis was performed to estimate the net clinical benefit of the predictive model.

## 3 Results

### 3.1 Patient characteristics

In this study, 183 GC patients aged 30 to 80 years (mean age: 60.65 ± 9.71 years, 146 males and 37 females) were recruited, with 70 serosal invasion-positive cases (T4 stage) and 113 serosal invasion-negative cases (T1–T3 stage).Patients were randomly divided into training (n = 128, median age 62 [IQR: 54–68]) and validation (n = 55, mean age 61.66 ± 9.85) sets at a 7:3 ratio. The training set included 49 serosal-positive and 79 serosal-negative cases, while the validation set contained 21 serosal-positive and 34 serosal-negative cases.

Clinical and CT characteristics of both cohorts are summarized in **Table 1**. No significant differences were observed between the training and validation sets in terms of sex, age, tumor location, ulceration, mucosal line continuity, serosal stranding, invasive growth pattern, lesion thickness, presence of enlarged lymph nodes, or CA724/CA199 levels (all p-values ranging from 0.098 to 0.991). The proportion of T4 cases was comparable between the training (38.3%, 49/128) and validation (38.2%, 21/55) sets, with no statistically significant difference.

**Table 1:** Baseline demographic, clinical, and CT characteristics of the training and validation sets.

Characteristic	Training set (N = 128)	Validation set (N = 55)	p-value
Age (years)	62 (54, 68)	61.66 ± 9.85	0.465
Gender, n (%)			0.098
Female	30 (23.4%)	7 (12.7%)	
Male	98 (76.6%)	48 (87.3%)	
CA199 (U/mL)	12.64 (6.71, 30.14)	11.66 (6.22, 37.74)	0.798
CA724 (U/mL)	3.30 (1.26, 9.71)	3.88 (1.44, 8.53)	0.723
Tumor Location, n (%)			0.641
Antrum	51 (39.8%)	23 (41.8%)	
Body	55 (43.0%)	20 (36.4%)	
Cardia	22 (17.2%)	12 (21.8%)	
Infiltrative Growth, n (%)			0.937
No	69 (53.9%)	30 (54.5%)	
Yes	59 (46.1%)	25 (45.5%)	
Ulceration, n (%)			0.756
No	55 (43.0%)	25 (45.5%)	
Yes	73 (57.0%)	30 (54.5%)	
Mucosal Line Disruption, n (%)			0.662
No	40 (31.3%)	19 (34.5%)	
Yes	88 (68.8%)	36 (65.5%)	
Serosal Stranding, n (%)			0.963
No	74 (57.8%)	32 (58.2%)	
Yes	54 (42.2%)	23 (41.8%)	
Lymph Node Enlargement, n (%)			0.991
No	86 (67.2%)	37 (67.3%)	
Yes	42 (32.8%)	18 (32.7%)	
Lesion Thickness (cm)	1.70 (1.30, 2.00)	1.60 (1.40, 2.00)	0.774
Serosal Invasion, n (%)			0.990
Negative (T1–T3)	79 (61.7%)	34 (61.8%)	
Positive (T4)	49 (38.3%)	21 (38.2%)	

Data are presented as mean ± standard deviation, median (interquartile range), or number (percentage), as appropriate. p-values in bold indicate statistical significance (p < 0.05).

### 3.2 Clinical features

Comparisons of clinical and CT characteristics between the two groups are detailed in **Tables 2** and **3**.

**Table 2:** Association of baseline clinical and CT characteristics with serosal invasion in the training cohort.

Characteristic	T1–T3 (n = 79)	T4 (n = 49)	p-value
Age (years)	59.43 ± 9.17	64 (55, 70)	0.098
Gender, n (%)			0.280
Female	16 (20.3%)	14 (28.6%)	
Male	63 (79.7%)	35 (71.4%)	
CA199, n (%)			0.042
Normal	68 (86.1%)	35 (71.4%)	
Abnormal	11 (13.9%)	14 (28.6%)	
CA724, n (%)			<0.001
Normal	66 (83.5%)	20 (40.8%)	
Abnormal	13 (16.5%)	29 (59.2%)	
Tumor Location, n (%)			0.243
Non-Cardia	63 (79.7%)	43 (87.8%)	
Cardia	16 (20.3%)	6 (12.2%)	
Infiltrative Growth Pattern, n (%)			0.007
Absent	50 (63.3%)	19 (38.8%)	
Present	29 (36.7%)	30 (61.2%)	
Ulceration, n (%)			0.728
Absent	33 (41.8%)	22 (44.9%)	
Present	46 (58.2%)	27 (55.1%)	
Mucosal Line Disruption, n (%)			0.091
Absent	29 (36.7%)	11 (22.4%)	
Present	50 (63.3%)	38 (77.6%)	
Serosal Stranding, n (%)			0.001
Absent	55 (69.6%)	19 (38.8%)	
Present	24 (30.4%)	30 (61.2%)	
Lymph Node Enlargement, n (%)			0.721
Absent	54 (68.4%)	32 (65.3%)	
Present	25 (31.6%)	17 (34.7%)	
Lesion Thickness (cm)	1.50 (1.30, 1.90)	1.80 (1.55, 2.35)	**0.004**
Rad-score	0.30 (0.15, 0.56)	0.67 (0.48, 0.83)	**<0.001**

Data are presented as mean ± standard deviation, median (interquartile range), or number (percentage), as appropriate. p-values in bold indicate statistical significance (p < 0.05).

**Table 3:** Association of baseline characteristics, clinical and CT features with serosal invasion status in the validation cohort.

Characteristic	T1–T3 (n = 34)	T4 (n = 21)	p-value
Age (years)	62.50 ± 10.40	60.29 ± 8.96	0.423
Gender, n (%)			0.491
Female	3 (8.8%)	4 (19.0%)	
Male	31 (91.2%)	17 (81.0%)	
CA199, n (%)			0.981
Normal	26 (76.5%)	16 (76.2%)	
Abnormal	8 (23.5%)	5 (23.8%)	
CA724, n (%)			0.004
Normal	26 (76.5%)	8 (38.1%)	
Abnormal	8 (23.5%)	13 (61.9%)	
Tumor Location, n (%)			0.956
Non-Cardia	26 (76.5%)	17 (81.0%)	
Cardia	8 (23.5%)	4 (19.0%)	
Infiltrative Growth Pattern, n (%)			0.418
Absent	20 (58.8%)	10 (47.6%)	
Present	14 (41.2%)	11 (52.4%)	
Ulceration, n (%)			0.418
Absent	14 (41.2%)	11 (52.4%)	
Present	20 (58.8%)	10 (47.6%)	
Mucosal Line Disruption, n (%)			0.188
Absent	14 (41.2%)	5 (23.8%)	
Present	20 (58.8%)	16 (76.2%)	
Serosal Stranding, n (%)			0.003
Absent	25 (73.5%)	7 (33.3%)	
Present	9 (26.5%)	14 (66.7%)	
Lymph Node Enlargement, n (%)			0.064
Absent	26 (76.5%)	11 (52.4%)	
Present	8 (23.5%)	10 (47.6%)	
Lesion Thickness (cm)	1.75 (1.40, 2.00)	1.65 ± 0.45	0.335
Rad-score	0.28 (0.14, 0.55)	0.66 (0.47, 0.76)	**<0.001**

Data are presented as mean ± standard deviation, median (interquartile range), or number (percentage), as appropriate. p-values in bold indicate statistical significance (p < 0.05).

### 3.3 Construction and validation of the radiomics model

A total of 2212 radiomics features were extracted per phase. Following consistency testing, features with intra- or inter-group ICC < 0.9 were excluded, retaining 1683 (AP), 1598 (VP), and 1564 (DP) stable radiomic features. Linear correlation analysis (threshold = 0.8) and L1-based feature selection were applied. Using logistic regression, single-phase models were constructed with 7 (AP), 9 (VP), and 8 (DP) features, respectively. AUC values for each model are shown in **Table 4**.

**Table 4:** Performance of single-phase, multi-phase for predicting serosal invasion in the training and validation sets.

Metric	AP model	VP model	DP model	AP+VP model	AP+DP model	VP+DP model	AP+VP+DP model
Training Set							
AUC	0.8241	0.7742	0.7706	0.8450	0.8256	0.7554	0.8460
Accuracy	0.6953	0.6797	0.6328	0.7656	0.7266	0.6562	0.7344
Sensitivity	0.7551	0.7143	0.6531	0.7755	0.7347	0.7143	0.7551
Specificity	0.6582	0.6582	0.6203	0.7595	0.7215	0.6203	0.7215
Validation Set							
AUC	0.7157	0.6373	0.6261	0.6765	0.8179	0.6625	0.7353
Accuracy	0.6364	0.6000	0.6182	0.6000	0.7091	0.6000	0.7091
Sensitivity	0.6667	0.6667	0.7619	0.7619	0.7619	0.6190	0.7143
Specificity	0.6176	0.5588	0.5294	0.5000	0.6765	0.5882	0.7059

AP, arterial phase; VP, venous phase; DP, delayed phase; AUC, area under the receiver operating characteristic curve.

In the training set, single-phase AP, VP, and DP models achieved AUCs of 0.8241, 0.7742, and 0.7706, respectively, indicating good performance. Corresponding validation set AUCs were 0.7157, 0.6373, and 0.6261. Two-phase combined models (AP+VP, AP+DP, VP+DP) yielded training AUCs of 0.845, 0.8256, and 0.7554, and validation AUCs of 0.6765, 0.8179, and 0.6625. The three-phase combined model (AP+VP+DP) achieved AUCs of 0.846 (training) and 0.7353 (validation).

Delong’s test was used for pairwise comparisons between models (**Table 5**). No significant inter-model differences were detected in the training and validation sets following Holm-Bonferroni correction (**Supplementary Table 1**).

**Table 5:** Delong test comparing the AUC values of eight models for predicting serosal invasion in the training and validation sets.

Metric	AP model	VP model	DP model	AP+VP model	AP+DP model	VP+DP model	AP+VP+DP model
Training Set							
AP Model	–	0.3631	0.3376	0.6982	0.9750	0.2322	0.6579
VP Model	0.3631	–	0.9439	0.1902	0.3487	0.7558	0.1782
DP Model	0.3376	0.9439	–	0.1762	0.3241	0.8042	0.1650
AP+VP Model	0.6982	0.1902	0.1762	–	0.7055	0.1144	0.9831
AP+DP Model	0.9750	0.3487	0.3241	0.7055	–	0.0385	0.3751
VP+DP Model	0.2322	0.7558	0.8042	0.1144	0.0385	–	0.0133
AP+VP+DP Model	0.6579	0.1782	0.1650	0.9831	0.3751	0.0133	–
Validation Set							
AP Model	–	0.4637	0.3931	0.6752	0.2632	0.6169	0.8415
VP Model	0.4637	–	0.9121	0.7164	0.0682	0.8226	0.3499
DP Model	0.3931	0.9121	–	0.6340	0.0476	0.7417	0.2878
AP+VP Model	0.6752	0.7164	0.6340	–	0.1277	0.8962	0.5534
AP+DP Model	0.2632	0.0682	0.0476	0.1277	–	0.0536	0.0608
VP+DP Model	0.6169	0.8226	0.7417	0.8962	0.0536	–	0.3549
AP+VP+DP Model	0.8415	0.3499	0.2878	0.5534	0.0608	0.3549	–

The values represent p-values from the Delong test for pairwise comparison of AUCs between models. AP, arterial phase; VP, venous phase; DP, delayed phase; NOMO, nomogram.

Based on comprehensive evaluation of AUC, accuracy (ACC), sensitivity (SEN), and specificity (SPE), the AP+DP model demonstrated superior and consistent performance across both sets (training AUC: 0.8256; validation AUC: 0.8179; training ACC/SEN/SPE: 0.7266/0.7347/0.7215; validation ACC/SEN/SPE: 0.7091/0.7619/0.6765). Thus, the AP+DP model was selected for integration with CT and clinical features in the subsequent nomogram construction.

AP+DP Model Development:

A total of 2212 features were initially extracted from each of the AP and DP phases. After ICC-based filtering (< 0.9 excluded), 1683 (AP) and 1564 (DP) features remained. Following removal of 650 redundant features and correlation-based filtering (r > 0.8), 112 features were retained. L1 regularization (C = 0.045) was applied, selecting 9 features (6 from AP, 3 from DP; see **Supplementary Table 2**; **Figure 3**). A logistic regression model was trained using these features.

**Figure 3:**
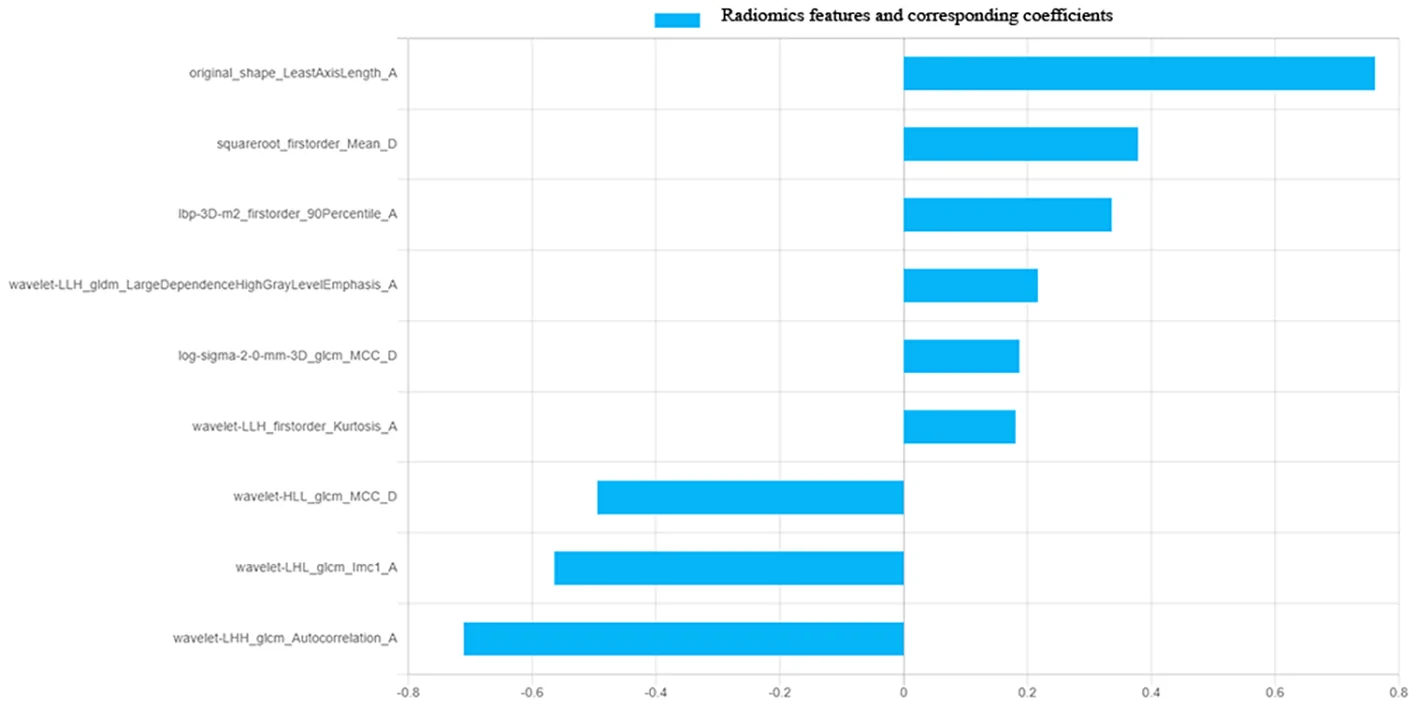
Radiomic features and their corresponding coefficients selected by the AP+DP model.

Rad-scores were significantly higher in serosal-positive than serosal-negative patients in both the training set (0.67 [0.48, 0.83] vs. 0.30 [0.15, 0.56], p < 0.001) and validation set (0.66 [0.47, 0.76] vs. 0.28 [0.14, 0.55], p < 0.001). The model achieved AUCs of 0.8256 (95% CI: 0.7546–0.8966) in training and 0.8179 (95% CI: 0.7063–0.9295) in validation.

### 3.4 Construction and validation of the combined predictive model

Univariate logistic regression identified the following potential predictors of serosal invasion: Rad-score, invasive growth, lesion thickness, CA199, CA724, and serosal stranding. Multivariate analysis confirmed CA724, serosal stranding, and Rad-score as independent predictors (**Table 6**). The clinical model was constructed based on CA72-4 and serosal stranding alone.

**Table 6:** Univariate and multivariate logistic regression analysis of predictive factors for serosal invasion.

Variable	Univariate analysis	p-value	Multivariate analysis	p-value
	OR		OR	
Age	1.023	0.246		
Gender	0.635	0.282		
CA199	2.473	0.046*	0.633	0.751
CA724	7.362	<0.001*	4.810	0.002*
Tumor Location	0.549	0.248		
Infiltrative Growth Pattern	2.722	0.008*	0.559	0.294
Ulceration	0.880	0.728		
Mucosal Line Disruption	2.004	0.094		
Serosal Stranding	3.618	0.001*	2.971	0.031*
Lymph Node Enlargement	1.147	0.721		
Lesion Thickness	1.976	0.021*	0.813	0.599

*indicates statistical significance (p < 0.05). Variables marked with * in the multivariate analysis were identified as independent predictors and were incorporated into the final nomogram model.

A nomogram incorporating these three factors was developed (**Figure 4**). The predictive performances of the AP+PD model, clinical model, and combined model are summarized in **Table 7**, and the ROC curves of each model are presented in **Figure 5**.

**Figure 4:**
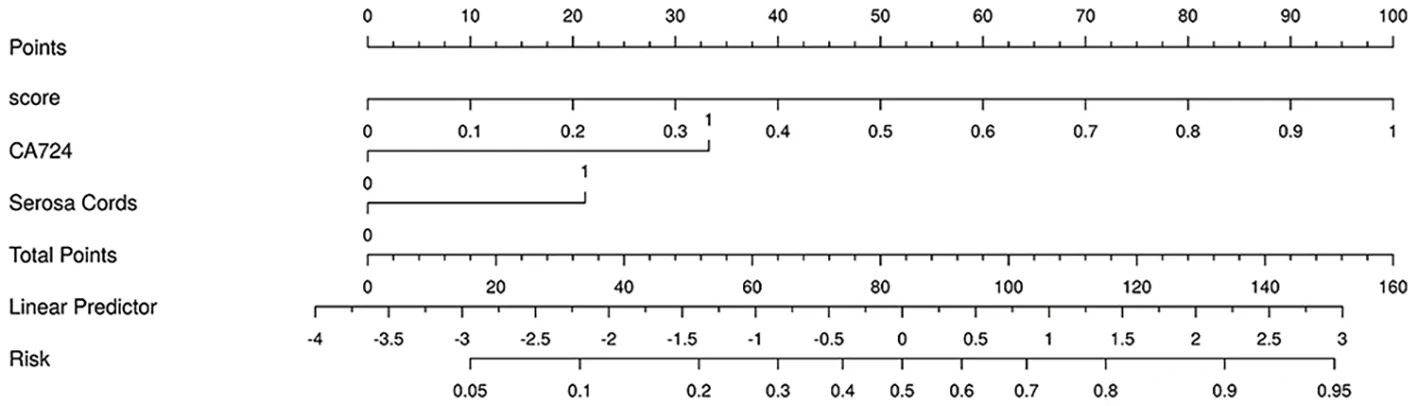
The nomogram incorporates Rad-score, CA72-4, and serosal stranding. Each predictor is assigned a point value based on its contribution to the model. The total points are summed and correspond to the predicted probability of serosal invasion on the bottom axis. This tool enables individualized preoperative risk estimation for gastric cancer patients.

**Table 7:** Performance of AP+DP model, clinical model and combined model in the training and validation sets.

Model	Set	AUC	Accuracy	Sensitivity	Specificity
AP+DP	Train	0.8256	0.7266	0.7347	0.7215
	Vol	0.8179	0.7091	0.7619	0.6765
Clinical	Train	0.7738	0.7422	0.5918	0.8354
	Vol	0.7654	0.7091	0.619	0.7647
combined model	Train	0.8626	0.7734	0.7551	0.7848
	Vol	0.8641	0.7636	0.8095	0.7353

**Figure 5:**
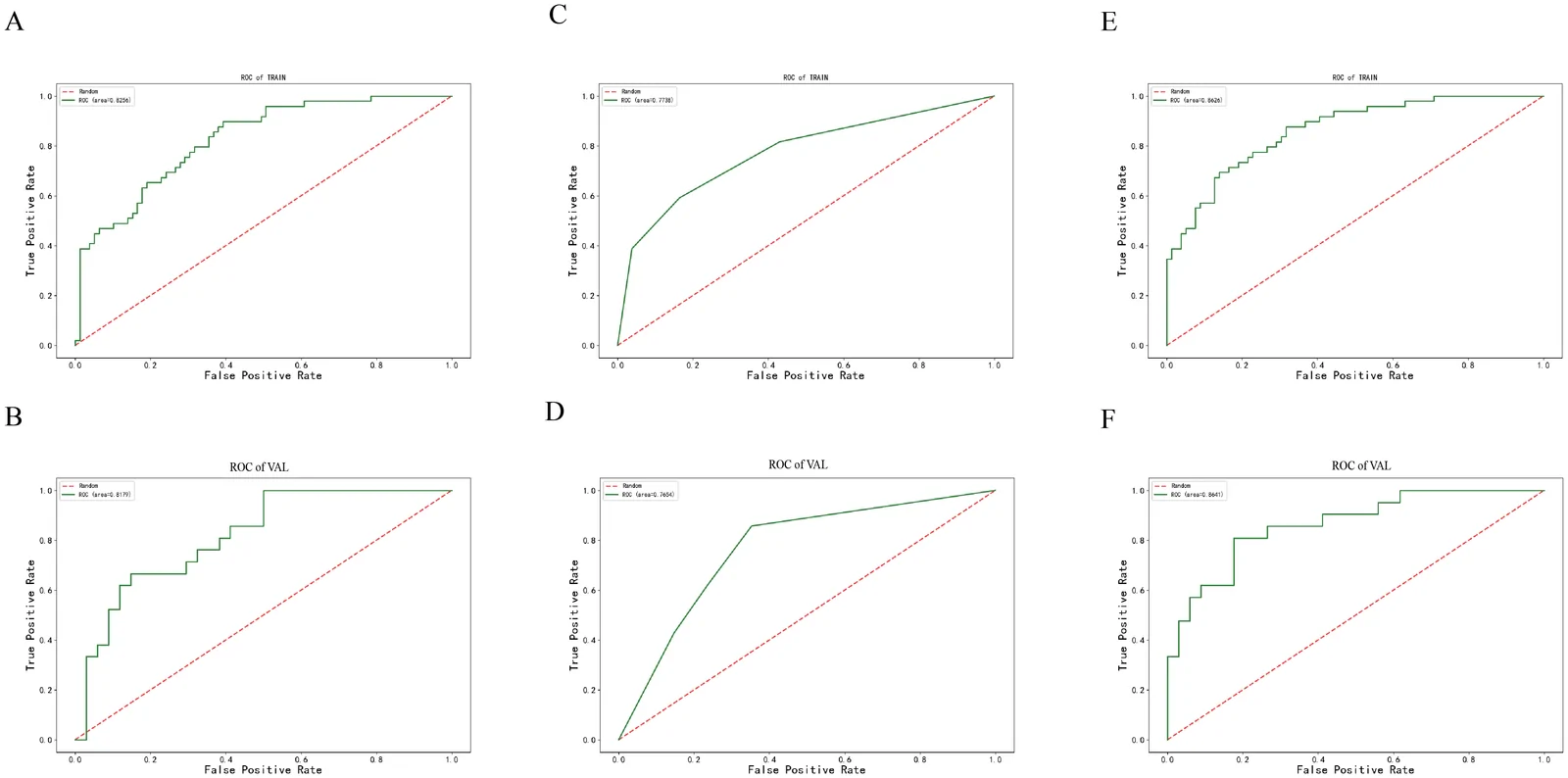
**(A, C, E)** present ROC curves of the AP+DP model, clinical model, and combined-nomogram model in the training cohort, whereas **(B, D, F)** show their corresponding curves in the validation cohort.

Delong’s test revealed that the combined model exhibited a statistically significant difference relative to the clinical model in the training set(p = 0.0017, adjusted p-value 0.0051). No significant differences were found in the following comparisons: the combined model versus the clinical model in the validation set(p = 0.0272, adjusted p-value 0.0816), the nomogram versus the AP+DP model in the training set (p = 0.446, adjusted p-value 0.446) and validation set (p = 0.5433, adjusted p-value 0.5433), as well as the A+D model versus the clinical model in both the training and validation sets(p = 0.3537(adjusted p-value 0.7074), p = 0.5397(adjusted p-value 1.0794)).

Calibration curves and the Hosmer-Lemeshow goodness-of-fit test were performed to evaluate the agreement between predicted and actual outcomes of the combined nomogram (**Figure 6**). The Brier scores were 0.155 (training set), and 0.152 (validation set), reflecting good predictive accuracy. Satisfactory goodness-of-fit was observed in the training set (Χ2= 10.7623, p = 0.2155), validation set (Χ2= 7.3682, p = 0.4975), indicating high consistency between model-predicted results and actual observations. Decision-curve analysis (DCA) revealed that both the combined nomogram and the clinical score yielded favorable net benefit across most threshold probabilities (**Figure 7**). Clinical intervention guided by the combined nomogram could benefit patients with gastric cancer in the training and validation sets.

**Figure 6:**
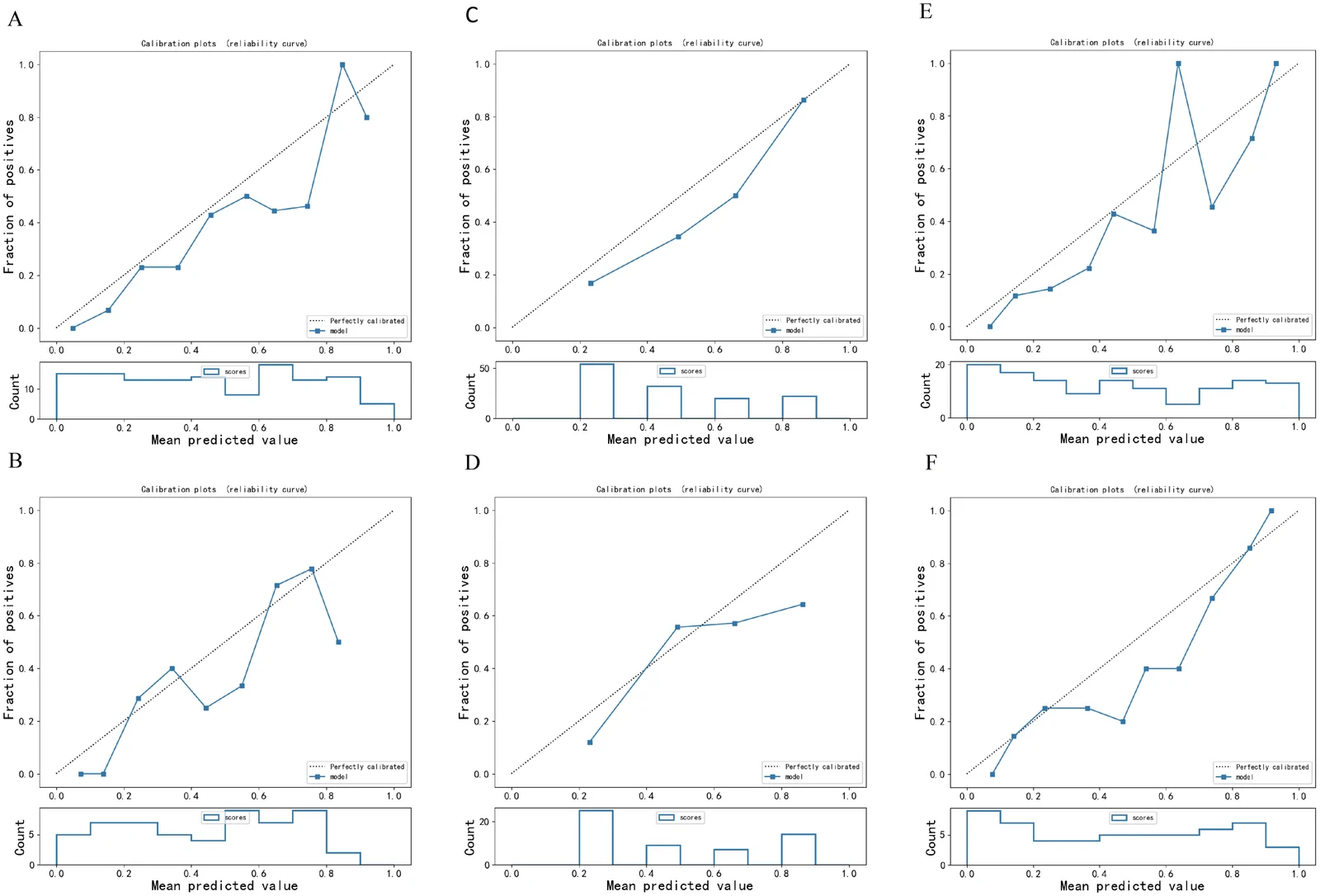
**(A, C, E)** present calibration curves of the AP+DP, clinical, and combined models in the training cohort, whereas **(B, D, F)** show their corresponding curves in the validation cohort. The x-axis represents the predicted probability of event occurrence generated by each model, and the y-axis indicates the actual observed incidence of the event. The grey 45° line serves as the reference line, representing perfect agreement between predicted and actual outcomes. The solid blue line denotes the fitted calibration curve corresponding to observed values. A closer proximity of the blue solid line to the grey reference line indicates better calibration performance of the model.

**Figure 7:**
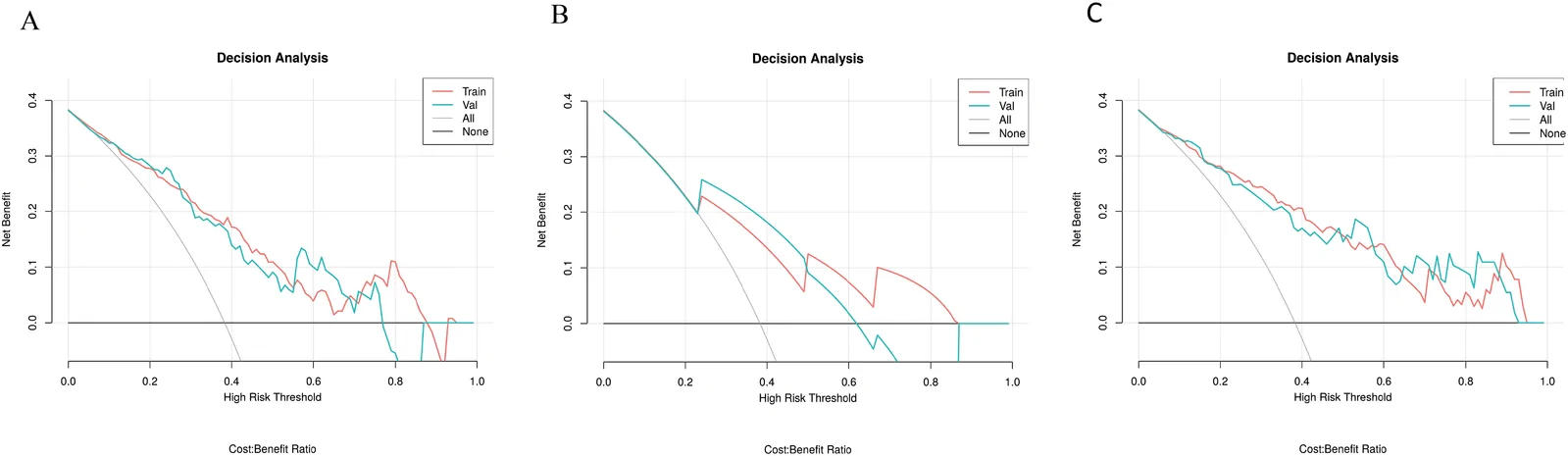
**(A–C)** present DCA curves of the AP+DP, clinical, and combined models, respectively. The x-axis shows the threshold probability, and the y-axis represents the net benefit. Across most threshold probability ranges in both the training and validation cohorts, the AP+DP model, clinical model, and combined model provided higher net benefit than the treat-all and treat-none strategies. The combined-nomogram exhibited the best performance.

## 4 Discussion

In this study, we developed seven radiomics models based on multiphase contrast-enhanced CT images. Among these, the AP+DP model demonstrated the best and most stable performance in both training and validation sets, showing good predictive capability. Furthermore, a combined nomogram model incorporating CA724 and serosal stranding was constructed, which outperformed the radiomics-only model with improved AUC values in both sets. This approach offers valuable assistance in accurately assessing serosal invasion preoperatively to inform further treatment decisions.

The NCCN guidelines recommend neoadjuvant chemotherapy (NAC) for patients with T4 advanced GC to reduce tumor stage, increase the R0 resection rate, and improve prognosis (6). Accurate preoperative staging of GC is critical for selecting appropriate treatment strategies. Studies have shown that preoperative NAC can reduce mortality in GC patients, underscoring the importance of precise evaluation of serosal invasion (27). However, the reported accuracy of CT-based T staging varies widely, with diagnostic accuracy for serosal invasion ranging from 55.9% to 90.8%. Therefore, there is a clear need for more accurate and reliable assessment methods.

Radiomics is a big data-based post-processing technique that objectively extracts vast amounts of information beyond human visual perception. It has been widely applied in medical fields, particularly in oncology. Quantitative image features based on intensity, shape, size, volume, and texture provide insights into tumor phenotype and microenvironment. These features, combined with other data, can be correlated with clinical outcomes to support evidence-based clinical decision-making (28). Studies indicate that tumors exhibit heterogeneity at tissue, cellular, genetic, and phenotypic levels, including spatial variations in cellular density, angiogenesis, and necrosis. Such heterogeneity may be associated with aggressive biological behavior (29, 30). Contrast-enhanced scans effectively reveal tumor vascularity and enhancement heterogeneity (31). Therefore, we utilized radiomics based on enhanced CT to extract subvisual image features to aid clinical decision-making. Existing studies on preoperative prediction of serosal invasion vary in phase selection and segmentation methods (2D-ROI vs. 3D-VOI). A key objective of this study was to determine which phase or combination of phases yields the most effective and stable model for predicting serosal invasion.

We found that the AP+DP model performed best among all single and combined phase models, achieving AUC, ACC, SEN, and SPE values of 0.8256, 0.7266, 0.7347, and 0.7215 in the training set, and 0.8179, 0.7091, 0.7619, and 0.6765 in the validation set, respectively. This model utilized nine features, six from the arterial phase and three from the delayed phase, including one morphological feature (Shape), three First Order features, four GLCM features, and one GLDM feature. The highest-weighted feature was shape_Least Axis Length from the arterial phase, which represents the minor axis length of the ellipsoid enclosing the ROI. In 2D segmentation, this value would be zero. In our study, the serosal invasion group had a larger least axis length than the non-invasion group. Previous research on predicting invasiveness in papillary thyroid carcinoma also found significant differences in this feature between invasive and non-invasive groups (32), where invasiveness was defined by capsular or extrathyroidal invasion—consistent with our findings. Similarly, the shape_Least Axis Length feature has been implicated in predicting postoperative grading in prostate cancer (associated with aggressiveness), complementing texture features in predictive models (33). These studies, along with ours, highlight the importance of this feature and underscore the necessity of using whole-lesion VOI for assessing serosal invasion.

Other arterial phase features included two First Order features-90 Percentile and Kurtosis-which are voxel-based. The 90th percentile is sensitive to subtle internal changes within the lesion, indicating regions of lower cellular density (e.g., necrosis). Kurtosis measures the “peakedness” of the voxel value distribution relative to a normal distribution, reflecting tissue heterogeneity and complexity. Lower kurtosis indicates higher heterogeneity and complexity, suggesting that more heterogeneous lesions are more likely to exhibit serosal invasion. The arterial phase is known to reflect blood supply and functional capillary density in GC (31), which likely differs between serosal-invasive and non-invasive tumors, with the former possibly having higher perfusion. Previous studies predicting T3 and T4a staging in gastroesophageal junction adenocarcinoma also incorporated these two features (24).

Additionally, one GLDM feature and two GLCM features from the arterial phase, which are based on paired voxels and describe relationships between adjacent voxels, were included. GLDM_Large Dependence High Gray Level Emphasis (LDHGLE) reflects the joint distribution of high-gray-level pixel dependencies. Higher LDHGLE values indicate that high-intensity voxels are more likely to be adjacent (34), suggesting that serosal-invasive tumors exhibit higher heterogeneity on arterial phase images. GLCM features reflect the joint probability distribution of pixel pairs, capturing comprehensive information on grayscale direction, distance, and amplitude, thereby aiding in the analysis of local heterogeneity. A higher GLCM_IMC1 indicates greater tissue homogeneity. Our model showed that non-invasive groups had higher values than invasive groups, implying relatively higher homogeneity in non-invasive lesions. Autocorrelation measures texture fineness and coarseness. Higher values in measurements of low-gray-level distribution indicate a greater concentration of low-intensity values in the image, which corresponds to darker areas in gastric CT images.

Besides the six arterial phase features, the model also incorporated three delayed phase features: one First Order feature (firstorder_Mean) and two glcm_MCC features derived from different image preprocessing methods. Mean represents the average grayscale intensity within the ROI. Higher values in the serosal invasion group may be related to higher voxel values in cellular regions, resulting in an overall higher lesion intensity. The other two features, glcm_MCC (Maximum Correlation Coefficient), measure texture complexity. We found that the invasive group had lower MCC values, indicating greater spatial heterogeneity (lower correlation between voxels) in their CT images.

The strong performance of the AP+DP model highlights the arterial phase as the most contributory phase for predicting serosal invasion, underscoring its importance. We speculate that since GC originates from gastric mucosal epithelium and causes wall thickening with moderate to marked enhancement in early stages, advanced GC often shows progressive enhancement from mucosa to serosa. The arterial phase provides information on blood supply and functional capillary density (31), supporting this view. Meanwhile, the inclusion of three delayed phase features also contributes to the model. Studies have shown that the enhancement pattern of GC correlates with pathological factors such as cell differentiation, amount of fibrous stroma, and infiltration pattern. Ulcerated tumors often contain varying degrees of fibrous tissue, with most lesions showing weak enhancement in arterial and portal phases but peak enhancement in the delayed phase (35). Approximately 55% of tumors in our study were ulcerated, which may explain the clearer delineation in delayed phases. Furthermore, peak enhancement in GC varies by histological type: differentiated types peak in arterial or portal phases, while undifferentiated types peak in the delayed phase (36). Histological type is one of the most important factors related to aggressiveness and prognosis in GC (37). Poorly differentiated types are often more aggressive, and serosal invasion is a manifestation of this aggressiveness. Based on our results, redundant overlapping features may coexist across the arterial, portal venous and delayed phases. Furthermore, the portal venous phase yields numerous ambiguous intermediate enhancement signals. Diffuse gastric wall enhancement in this phase leads to simultaneous enhancement of normal gastric wall, tumor and peritumoral inflammatory tissue, diminishing tissue contrast. Strands from T3 inflammation and T4 tumor infiltration are difficult to differentiate on portal venous phase images, likely inducing false positives and impairing diagnostic performance. This observation may correlate with the pathological profiles of our cohort, including tumor differentiation, Lauren classification and fibrotic proportion. Further validation using larger sample sizes or external cohorts is warranted.

We further integrated clinical and CT features to construct a nomogram model. Univariate and multivariate logistic regression identified CA724 and serosal stranding as independent predictors of serosal invasion. The inclusion of CA724 is consistent with previous studies (22). Research by Cidon and Bustamante showed that preoperative serum CA724 levels have optimal predictive value for advanced GC (38). Chen et al. found that CA724 is the most relevant serum tumor marker for GC (39). Many studies have reported rising serum CA724 levels with advancing GC stage. A prospective study found a significant difference in preoperative CA724 positivity between early (stage I/II) and advanced (stage III/IV) GC patients (0% vs. 71.8%) (40). Jixian et al., in a study of 389 GC patients, also reported that serum CA724 levels correlated positively with TNM stage (p < 0.05) and histological grade (p < 0.05), suggesting its utility in predicting invasion and metastasis (41). Although both CA199 and CA724 were included in univariate analysis, only CA724 remained significant in multivariate analysis, indicating that CA199 is a predictor but not an independent one, possibly due to correlations with other factors.

We also found statistically significant differences in invasive growth pattern and lesion thickness between serosal-negative and serosal-positive groups in the training set (p < 0.05). Infiltrative growth is associated with greater aggressiveness and higher likelihood of serosal invasion. Previous studies have identified perigastric stranding as an independent predictor for distinguishing T3 from T4a tumors in gastroesophageal junction cancer (24). Our model confirmed serosal stranding as an independent predictor. Some studies also include peritumoral fat density as a factor (42); however, we observed overlap between serosal stranding and peritumoral fat density increase during data collection, and due to potential collinearity, the latter was not included.

Ultimately, our study demonstrates that the arterial plus delayed phase radiomics model outperformed other phase combinations. Interestingly, using more phases did not necessarily improve performance. We also confirmed that whole-lesion VOI is essential, as the highest-weighted feature in our model could only be derived from 3D-VOI. The nomogram integrating the AP+DP radiomics signature with serosal stranding and CA72-4 achieved better performance than the radiomics-only model. Furthermore, the combined model significantly outperformed the clinical model in the training set, with a marked improvement in diagnostic performance Although the combined nomogram achieved numerically higher AUCs than the radiomics-only model, the differences did not reach statistical significance, likely due to limited sample size. Nonetheless, the nomogram offers improved clinical interpretability and practical utility, as supported by calibration and decision-curve analyses. Further validation in larger cohorts is warranted to confirm its added value.

Our study has several limitations: First, its retrospective nature may introduce selection bias. Second, as a single-center study with a relatively small sample size and lacking external validation, future multicenter studies are needed to validate the model’s generalizability. Third, although we used semi-automatic segmentation with ICCs to ensure reproducibility, manual delineation of poorly defined tumors remains challenging. Automated segmentation methods would save time and improve consistency.

## 5 Conclusion

The radiomics model based on arterial and delayed phase contrast-enhanced CT images shows promise in predicting serosal invasion in gastric cancer. Furthermore, the incorporation of two independent clinical predictors-CA724 levels and serosal stranding-into a nomogram model further improved diagnostic performance, which was significantly superior to the clinical model in the training set, offering valuable support for preoperative clinical decision-making in GC patients.

## Data Availability

The raw data supporting the conclusions of this article will be made available by the authors, without undue reservation.

## References

[1] Sung H Ferlay J Siegel RL Laversanne M Soerjomataram I Jemal A . Global cancer statistics 2020: GLOBOCAN estimates of incidence and mortality worldwide for 36 cancers in 185 countries. CA Cancer J Clin. (2021) 71:209–49. doi: 10.3322/caac.21660 33538338

[2] Ajani JA D'Amico TA Bentrem DJ Chao J Cooke D Corvera C . Gastric cancer, version 2.2022, NCCN clinical practice guidelines in oncology. J Natl Compr Canc Netw. (2022) 20:167–92. doi: 10.6004/jnccn.2022.0008 35130500

[3] Kim JM Jung H Lee JS Lee HH Song KY Park CH . Clinical implication of serosal change in pathologic subserosa-limited gastric cancer. World J Surg. (2012) 36:355–61. doi: 10.1007/s00268-011-1334-x 22146944

[4] Tokumitsu Y Yoshino S Iida M Yoshimura K Ueno T Hazama S . Intraoperative dissemination during gastrectomy for gastric cancer associated with serosal invasion. Surg Today. (2015) 45:746–51. doi: 10.1007/s00595-014-1005-2 25115929

[5] Rijken A Lurvink RJ Luyer MDP Nieuwenhuijzen GAP van Erning FN van Sandick JW . The burden of peritoneal metastases from gastric cancer: A systematic review on the incidence, risk factors and survival. J Clin Med. (2021) 10. doi: 10.3390/jcm10214882 34768402 PMC8584453

[6] Smyth EC Verheij M Allum W Cunningham D Cervantes A Arnold D . Gastric cancer: ESMO clinical practice guidelines for diagnosis, treatment and follow-up. Ann Oncol. (2016) 27:v38–49. doi: 10.1093/annonc/mdw350 27664260

[7] Hizawa K Iwai K Esaki M Matsumoto T Suekane H Iida M . Is endoscopic ultrasonography indispensable in assessing the appropriateness of endoscopic resection for gastric cancer? Endoscopy. (2002) 34:973–8. doi: 10.1055/s-2002-35851 12471541

[8] Joo I Lee JM Kim JH Shin CI Han JK Choi BI . Prospective comparison of 3T MRI with diffusion-weighted imaging and MDCT for the preoperative TNM staging of gastric cancer. J Magn Reson Imaging. (2015) 41:814–21. doi: 10.1002/jmri.24586 24677322

[9] Hasegawa S Yoshikawa T Shirai J Fujikawa H Cho H Doiuchi T . A prospective validation study to diagnose serosal invasion and nodal metastases of gastric cancer by multidetector-row CT. Ann Surg Oncol. (2013) 20:2016–22. doi: 10.1245/s10434-012-2817-1 23266583

[10] Kim TU Kim S Lee JW Lee NK Jeon TY Park DY . MDCT features in the differentiation of T4a gastric cancer from less-advanced gastric cancer: Significance of the hyperattenuating serosa sign. Br J Radiol. (2013) 86:20130290. doi: 10.1259/bjr.20130290 23873904 PMC3755399

[11] Fukagawa T Katai H Mizusawa J Nakamura K Sano T Terashima M . A prospective multi-institutional validity study to evaluate the accuracy of clinical diagnosis of pathological stage III gastric cancer (JCOG1302A). Gastric Cancer. (2018) 21:68–73. doi: 10.1007/s10120-017-0701-1 28194522

[12] Yoshikawa T Tanabe K Nishikawa K Ito Y Matsui T Kimura Y . Accuracy of CT staging of locally advanced gastric cancer after neoadjuvant chemotherapy: Cohort evaluation within a randomized phase II study. Ann Surg Oncol. (2014) 21:S385-9. doi: 10.1245/s10434-014-3615-8 24595801

[13] Gillies RJ Kinahan PE Hricak H . Radiomics: Images are more than pictures, they are data. Radiology. (2016) 278:563–77. doi: 10.1148/radiol.2015151169 26579733 PMC4734157

[14] Moon SH Kim J Joung JG Cha H Park WY Ahn JS . Correlations between metabolic texture features, genetic heterogeneity, and mutation burden in patients with lung cancer. Eur J Nucl Med Mol Imaging. (2019) 46:446–54. doi: 10.1007/s00259-018-4138-5 30145701

[15] Choi ER Lee HY Jeong JY Choi YL Kim J Bae J . Quantitative image variables reflect the intratumoral pathologic heterogeneity of lung adenocarcinoma. Oncotarget. (2016) 7:67302–13. doi: 10.18632/oncotarget.11693 27589833 PMC5341876

[16] Sala E Mema E Himoto Y Veeraraghavan H Brenton JD Snyder A . Unravelling tumour heterogeneity using next-generation imaging: Radiomics, radiogenomics, and habitat imaging. Clin Radiol. (2017) 72:3–10. doi: 10.1016/j.crad.2016.09.013 27742105 PMC5503113

[17] Huang H Xu F Chen Q Hu H Qi F Zhao J . The value of CT-based radiomics nomogram in differential diagnosis of different histological types of gastric cancer. Phys Eng Sci Med. (2022) 45:1063–71. doi: 10.1007/s13246-022-01170-y 36063347 PMC9747822

[18] Wang X Li C Fang M Zhang L Zhong L Dong D . Integrating No.3 lymph nodes and primary tumor radiomics to predict lymph node metastasis in T1-2 gastric cancer. BMC Med Imaging. (2021) 21:58. doi: 10.1186/s12880-021-00587-3 33757460 PMC7989204

[19] Guo Q Sun Q Bian X Wang M Dong H Yin H . Development and validation of a multiphase CT radiomics nomogram for the preoperative prediction of lymphovascular invasion in patients with gastric cancer. Clin Radiol. (2023) 78:e552-e9. doi: 10.1016/j.crad.2023.03.016 37117048

[20] Liu S He J Liu S Ji C Guan W Chen L . Radiomics analysis using contrast-enhanced CT for preoperative prediction of occult peritoneal metastasis in advanced gastric cancer. Eur Radiol. (2020) 30:239–46. doi: 10.2139/ssrn.3297887 31385045

[21] Chen Y Yuan F Wang L Li E Xu Z Wels M . Evaluation of dual-energy CT derived radiomics signatures in predicting outcomes in patients with advanced gastric cancer after neoadjuvant chemotherapy. Eur J Surg Oncol. (2022) 48:339–47. doi: 10.1016/j.ejso.2021.07.014 34304951

[22] Liu S Xu M Qiao X Ji C Li L Zhou Z . Prediction of serosal invasion in gastric cancer: Development and validation of multivariate models integrating preoperative clinicopathological features and radiographic findings based on late arterial phase CT images. BMC Cancer. (2021) 21:1038. doi: 10.1186/s12885-021-08672-0 34530755 PMC8447770

[23] Zheng L Zhang X Hu J Gao Y Zhang X Zhang M . Establishment and applicability of a diagnostic system for advanced gastric cancer T staging based on a faster region-based convolutional neural network. Front Oncol. (2020) 10:1238. doi: 10.3389/fonc.2020.01238 32850373 PMC7399625

[24] Chang X Guo X Li X Han X Li X Liu X . Potential value of radiomics in the identification of stage T3 and T4a esophagogastric junction adenocarcinoma based on contrast-enhanced CT images. Front Oncol. (2021) 11:627947. doi: 10.3389/fonc.2021.627947 33747947 PMC7968370

[25] Wang Y Liu W Yu Y Liu JJ Jiang L Xue HD . Prediction of the depth of tumor invasion in gastric cancer: Potential role of CT radiomics. Acad Radiol. (2020) 27:1077–84. doi: 10.1016/j.acra.2019.10.020 31761666

[26] Wang L Zhang Y Chen Y Tan J Wang L Zhang J . The performance of a dual-energy CT derived radiomics model in differentiating serosal invasion for advanced gastric cancer patients after neoadjuvant chemotherapy: Iodine map combined with 120-kV equivalent mixed images. Front Oncol. (2020) 10:562945. doi: 10.3389/fonc.2020.562945 33585186 PMC7874026

[27] Coccolini F Nardi M Montori G Ceresoli M Celotti A Cascinu S . Neoadjuvant chemotherapy in advanced gastric and esophago-gastric cancer. Meta-analysis of randomized trials. Int J Surg. (2018) 51:120–7. doi: 10.1016/j.ijsu.2018.01.008 29413875

[28] Attanasio S Forte SM Restante G Gabelloni M Guglielmi G Neri E . Artificial intelligence, radiomics and other horizons in body composition assessment. Quant Imaging Med Surg. (2020) 10:1650–60. doi: 10.21037/qims.2020.03.10 32742958 PMC7378090

[29] Hudler P . Challenges of deciphering gastric cancer heterogeneity. World J Gastroenterol. (2015) 21:10510–27. doi: 10.3748/wjg.v21.i37.10510 26457012 PMC4588074

[30] Kim JY Gatenby RA . Quantitative clinical imaging methods for monitoring intratumoral evolution. Methods Mol Biol. (2017) 1513:61–80. doi: 10.1007/978-1-4939-6539-7_6 27807831

[31] Chen XH Ren K Liang P Chai YR Chen KS Gao JB . Spectral computed tomography in advanced gastric cancer: Can iodine concentration non-invasively assess angiogenesis? World J Gastroenterol. (2017) 23:1666–75. doi: 10.3748/wjg.v23.i9.1666 28321168 PMC5340819

[32] Leitch K Halicek M Shahedi M Little JV Chen AY Fei B . Detecting aggressive papillary thyroid carcinoma using hyperspectral imaging and radiomic features. Proc SPIE Int Soc Opt Eng. (2022) 12033. doi: 10.1117/12.2611842 36798628 PMC9929637

[33] Ghezzo S Mapelli P Bezzi C Samanes Gajate AM Brembilla G Gotuzzo I . Role of [(68)Ga]Ga-PSMA-11 PET radiomics to predict post-surgical ISUP grade in primary prostate cancer. Eur J Nucl Med Mol Imaging. (2023) 50:2548–59. doi: 10.1007/s00259-023-06187-3 36933074

[34] van Griethuysen JJM Fedorov A Parmar C Hosny A Aucoin N Narayan V . Computational radiomics system to decode the radiographic phenotype. Cancer Res. (2017) 77:e104-e7. doi: 10.1158/0008-5472.can-17-0339 29092951 PMC5672828

[35] Tsurumaru D Miyasaka M Nishimuta Y Asayama Y Nishie A Kawanami S . Differentiation of early gastric cancer with ulceration and resectable advanced gastric cancer using multiphasic dynamic multidetector CT. Eur Radiol. (2016) 26:1330–7. doi: 10.1007/s00330-015-3938-2 26242583

[36] Tsurumaru D Miyasaka M Muraki T Nishie A Asayama Y Oki E . Histopathologic diversity of gastric cancers: Relationship between enhancement pattern on dynamic contrast-enhanced CT and histological type. Eur J Radiol. (2017) 97:90–5. doi: 10.1016/j.ejrad.2017.10.018 29153374

[37] Zheng HC Zheng YS Xia P Xu XY Xing YN Takahashi H . The pathobiological behaviors and prognosis associated with Japanese gastric adenocarcinomas of pure WHO histological subtypes. Histol Histopathol. (2010) 25:445–52. doi: 10.14670/HH-25.445 20183797

[38] Cidón EU Bustamante R . Gastric cancer: Tumor markers as predictive factors for preoperative staging. J Gastrointest Cancer. (2011) 42:127–30. doi: 10.1007/s12029-010-9161-0 20499209

[39] Chen XZ Zhang WK Yang K Wang LL Liu J Wang L . Correlation between serum CA724 and gastric cancer: Multiple analyses based on Chinese population. Mol Biol Rep. (2012) 39:9031–9. doi: 10.1007/s11033-012-1774-x 22752725

[40] Rossi G Ignatiadis M . Promises and pitfalls of using liquid biopsy for precision medicine. Cancer Res. (2019) 79:2798–804. doi: 10.1158/0008-5472.can-18-3402 31109952

[41] Jiexian J Xiaoqin X Lili D Baoguo T Ting S Xianwen Z . Clinical assessment and prognostic evaluation of tumor markers in patients with gastric cancer. Int J Biol Markers. (2013) 28:192–200. doi: 10.5301/jbm.5000023 23787496

[42] Yang L Sun J Yu X Li Y Li M Liu J . Diagnosis of serosal invasion in gastric adenocarcinoma by dual-energy CT radiomics: Focusing on localized gastric wall and peritumoral radiomics features. Front Oncol. (2022) 12:848425. doi: 10.3389/fonc.2022.848425 35387116 PMC8977467

