# Supplementary material for: Preoperative prediction of serosal invasion in gastric cancer using multiphase contrast-enhanced CT radiomics
Source: Front Oncol. 2026 Sep 15;16:1879678. doi: 10.3389/fonc.2026.1879678 (PMC13619398; doi:10.3389/fonc.2026.1879678)
Supplement: Supplementary file 1 [file Table1.docx]

Table S1 *p*‑values from DeLong’s test adjusted by the Holm‑Bonferroni method

|  | AP Model | VP Model | DP Model | AP+VP Model | AP+DP Model | VP+DP Model | AP+VP+DP Model |
| --- | --- | --- | --- | --- | --- | --- | --- |
|  |  |  |  |  |  |  |  |
| Training Set |  |  |  |  |  |  |  |
| AP Model |  | 3.631 | 4.0512 | 4.8874 | 1.95 | 3.2508 | 5.2632 |
| VP Model |  |  | 2.8317 | 2.853 | 3.8357 | 3.779 | 2.8512 |
| DP Model |  |  |  | 2.9954 | 4.2133 | 3.2168 | 2.97 |
| AP+VP Model |  |  |  |  | 4.233 | 2.1736 | 0.9831 |
| AP+DP Model |  |  |  |  |  | 0.77 | 3.3759 |
| VP+DP Model |  |  |  |  |  |  | 0.2793 |
| AP+VP+DP Model |  |  |  |  |  |  |  |
| Validation Set |  |  |  |  |  |  |  |
| AP Model |  | 5.1007 | 4.7172 | 4.7264 | 4.2112 | 5.5521 | 2.5245 |
| VP Model |  |  | 0.9121 | 4.2984 | 1.2276 | 3.2904 | 4.8986 |
| DP Model |  |  |  | 5.072 | 0.9996 | 3.7085 | 4.317 |
| AP+VP Model |  |  |  |  | 2.1709 | 1.7924 | 5.534 |
| AP+DP Model |  |  |  |  |  | 1.072 | 1.1552 |
| VP+DP Model |  |  |  |  |  |  | 4.6137 |
| AP+VP+DP Model |  |  |  |  |  |  |  |

Table S2 Radiomic Features Selected for the AP+DP Model

| **Radiomics Feature Names** | **Coefficient** | **Relative Weight** |
| --- | --- | --- |
| original_shape_LeastAxisLength_A | 0.7609 | 1.0000 |
| squareroot_firstorder_Mean_D | 0.3783 | 0.4972 |
| lbp-3D-m2_firstorder_90Percentile_A | 0.3358 | 0.4413 |
| wavelet-LLH_gldm_LargeDependenceHighGrayLevelEmphasis_A | 0.2166 | 0.2847 |
| log-sigma-2-0-mm-3D_glcm_MCC_D | 0.1866 | 0.2452 |
| wavelet-LLH_firstorder_Kurtosis_A | 0.1803 | 0.2370 |
| wavelet-HLL_glcm_MCC_D | -0.4943 | -0.6497 |
| wavelet-LHL_glcm_Imc1_A | -0.5639 | -0.7412 |
| wavelet-LHH_glcm_Autocorrelation_A | -0.7101 | -0.9333 |
